# Learning Outcome After Different Combinations of Seven Learning Activities in Basic Life Support on Laypersons in Workplaces: a Cluster Randomised, Controlled Trial

**DOI:** 10.1007/s40670-020-01160-3

**Published:** 2020-11-18

**Authors:** Helene Bylow, Thomas Karlsson, Margret Lepp, Andreas Claesson, Jonny Lindqvist, Leif Svensson, Johan Herlitz

**Affiliations:** 1 Department of Molecular and Clinical Medicine, Institute of Medicine, Sahlgrenska Academy, University of Gothenburg, Gothenburg, Sweden; 2 Health Metrics Unit, Institute of Medicine, Sahlgrenska Academy, University of Gothenburg, Gothenburg, Sweden; 3 Institute of Health and Care Sciences, Sahlgrenska Academy, University of Gothenburg, Gothenburg, Sweden; 4 Østfold University College, Halden, Norway; 5 School of Nursing and Midwifery, Griffith University, Brisbane, Australia; 6 Department of Medicine, Centre for Resuscitation Science, Karolinska Institute, Stockholm, Sweden; 7 Centre of Registers Västra Götaland, Gothenburg, Sweden; 8 Prehospen-Centre of Prehospital Research, Faculty of Caring Science, Work Life and Social Welfare, University of Borås, Borås, Sweden

**Keywords:** Basic life support, Learning activities, Learning outcome, Out-of-hospital cardiac arrest, Cardiopulmonary resuscitation, Automated external defibrillation

## Abstract

**Background:**

The goal for laypersons after training in basic life support (BLS) is to act effectively in an out-of-hospital cardiac arrest situation. However, it is still unclear whether BLS training targeting laypersons at workplaces is optimal or whether other effective learning activities are possible.

**Aim:**

The primary aim was to evaluate whether there were other modes of BLS training that improved learning outcome as compared with a control group, i.e. standard BLS training, six months after training, and secondarily directly after training.

**Methods:**

In this multi-arm trial, lay participants (*n* = 2623) from workplaces were cluster randomised into 16 different BLS interventions, of which one, instructor-led and film-based BLS training, was classified as control and standard, with which the other 15 were compared. The learning outcome was the total score for practical skills in BLS calculated using the modified Cardiff Test.

**Results:**

Four different training modes showed a significantly higher total score compared with standard (mean difference 2.3–2.9). The highest score was for the BLS intervention including a preparatory web-based education, instructor-led training, film-based instructions, reflective questions and a chest compression feedback device (95% CI for difference 0.9–5.0), 6 months after training.

**Conclusion:**

BLS training adding several different combinations of a preparatory web-based education, reflective questions and chest compression feedback to instructor-led training and film-based instructions obtained higher modified Cardiff Test total scores 6 months after training compared with standard BLS training alone. The differences were small in magnitude and the clinical relevance of our findings needs to be further explored.

**Trial Registration:**

ClinicalTrials.gov Identifier: NCT03618888. Registered August 07, 2018—Retrospectively registered, https://clinicaltrials.gov/ct2/show/NCT03618888

**Supplementary Information:**

The online version contains supplementary material available at 10.1007/s40670-020-01160-3.

## Introduction

Learning activities in basic life support (BLS) for laypersons must bridge the reality of an out-of-hospital cardiac arrest (OHCA) situation. It is urgent to alert the emergency medical service (EMS) and initiate high-quality cardiopulmonary resuscitation (CPR) and automated external defibrillation (AED) [1–5]. Medical and educational scientific guidelines in BLS are updated continuously to improve the outcome of cardiac arrest [1–4, 6, 7].

The learning outcome for training in BLS includes sufficient practical skills and theoretical knowledge. Experiential learning might be appropriate in order to understand the process of learning BLS. According to Kolb’s experiential learning theory, learning is individual and comprises four stages: (1) concrete experience, (2) reflective observation, (3) abstract conceptualisation and (4) active experimentation [8]. Different learning activities may create different conditions for learning [8].

Instructor-directed training compared with self-directed training and video-based instructions has been shown to be as good as instructions from the instructor alone [2, 9–13]. Peer learning, in which students interact with each other in order to learn, has also been shown to be effective [14]. Previous studies have reported that the quality of the chest compressions appears to be better when they are facilitated by an instructor compared with self-learning [15], with brief retraining within 6 months [16], in compression-only CPR [17] and when a feedback device for correct compressions was used [17–20]. In contrast, compressions may be too deep if a CPR feedback device is used [19, 21]. Moreover, the consideration of mastery learning, when the participant trains until mastery level is attained and checked by assessment, and deliberate practice, when learning is achieved by focusing deliberately on change to reach the goal, is recommended [2, 4, 22, 23]. In addition, learning can take the form of traditional BLS training, with all the information delivered at the same time, or it can be spaced out in the form of a low dose of training at high frequency [2, 4, 24]. Retraining for laypersons is recommended more often than once a year, as the quality decreases after 3 to 6 months [2]. Further, contextual learning with time for experience and reflection such as team training in scenarios with feedback appears to increase communication and learning in practical skills in BLS [2, 4]. Moreover, multi-media tools, such as mobile devices, video films and web-based platforms, constitute the current infrastructure and reflection on the learning process is essential [25, 26].

Globally, cardiovascular disease (CVD) is a major public health issue and an increased level of educated community members may increase cardiovascular health [5]. Workplaces in Sweden are subject to the Work Environment Act, which includes sufficient theoretical knowledge and practical skills in first aid and BLS for employees [27, 28]. Despite this, there is a lack of various learning activities to bridge an effective workplace-based learning to reach preparedness of an acute life-threatening situation. In addition, time for learning is needed so that the person really learns to perform the task.

There are scientific gaps in relation to educational interventions in BLS, including OHCA, CVD and the effectiveness of digital strategies [3, 4, 17, 26]. Little is known about various learning activities and blended learning in BLS targeting laypersons at workplaces. In two previous studies, we compared self-learning versus instructor-led learning and a preparatory web-based education versus no web-based education [29–31]. In this study with new analysis, we intended to cover some of the residual knowledge gap on different learning activities and compared 15 different combinations of seven learning activities with a standard BLS training. The primary aim of this study was thus to evaluate whether there were other modes of BLS training that improved learning outcome as compared with a control group, i.e. standard BLS training, 6 months after training. The secondary aim was to evaluate learning outcome directly after training. The hypotheses were that the learning activities, instructor-led training, a preparatory web-based education, chest compression feedback and reflective questions can increase learning outcome when compared to standard training in BLS.

## Materials and Methods

### Study Design

This study was an educational, multi-factorial intervention in BLS, CPR and AED for laypersons at workplaces in Sweden in 2014 to 2016. The design was a 16-arm parallel-group, experimental, cluster randomised, controlled trial (RCT) according to Reporting of Multi-Arm Parallel-Group Randomized Trials Extension of the CONSORT 2010 Statement [32] and checklist (Supplemental file 1). The trial compared 15 different interventions in BLS with a control group which was classified as standard, i.e. instructor-led and film-based BLS training. A formal power calculation was performed in two previous publications [29–31].

### Randomisation

Allocated laypersons were cluster randomised to one of 16 BLS training interventions using blocks of 25 individuals in 112 clusters calculated by the randomizer.org [33]. The generated interventions were delivered to the participants and the instructors by an independent co-ordinator at the central municipal and at the workplaces. The participants were only aware of their individual intervention. The BLS instructors were aware of the learning objective for the instructor-led intervention but not the study aim, trial design, randomisation or the overall interventions. According to the PROBE (Prospective Randomised Open Blinded End-Point Evaluation) design [34], the investigator (HB) was blinded to the randomisation of the participants, the training and the participants’ intervention at the assessment. The trial was registered at ClinicalTrials.gov (ID: NCT03618888).

### Study Population

The study population (*n* = 2623) was recruited from 84 workplaces in the community outside hospitals, in the south of Sweden. The criteria for inclusion were laypersons with no BLS training at all (CPR 35.5%, AED 79.6%) or without prior BLS training within 5 years or longer (CPR 64.5%, AED 20.4%) and 18 years of age or older. The criteria for exclusion were that potential candidates for participation were either healthcare professionals or participants who had not performed the retention test. The participants and workplaces were given both oral and written information and written informed consent was obtained from all the participants.

### Intervention

The BLS algorithm was according to the 2010 European Resuscitation Council (ERC) guidelines [35, 36]. Seven additional learning activities were explored in 16 different combinations, of which one was the control group. All the interventions included both theory and practice in BLS and the participants trained on a personal mini-manikin and a paperboard AED in a kit (Mini Anne Plus kit, Laerdal Medical, Stavanger, Norway). The participants self-assessed their practical skills level for the compressions by clicking for correct hand placement and depth and a raised chest for correct ventilation on the training manikin. The learning objective, which was the same for all the participants, was to train BLS according to guidelines and the BLS algorithm with the recognition of a victim in cardiac arrest, alerting the EMS, performing CPR (30 compressions and two ventilations) and using the AED. Half the interventions were self-directed, and the other half were instructor directed. For instructions, four self-learning groups were directed by a mobile application and 12 interventions were film based. The instructor-led training included involvement in a brief two-person mini-manikin OHCA scenario. Half the interventions included a preparatory web-based interactive education on stroke, acute myocardial infarction (AMI), OHCA, CPR, AED and healthy lifestyle factors. Half the interventions included three reflective essential questions put to the participants on calling for help, hand position for chest compressions and willingness to act in a real-life OHCA situation. In four of the interventions, a device for feedback on chest compression depth placed on the manikin’s chest was used. The duration of the instructor-led training was defined in the design of the study. The self-learning group could train for as long time as they wanted even if the instruction in the mobile application was 30 min and the instruction in the film was 60 min. Therefore, the exact duration for the training in all the learning activities was not defined. The approximate times for the duration of each learning material are presented in Table 1. A detailed description of the interventions is presented in Supplementary file 2.

**Table 1:** Characteristics of the participants according to intervention in BLS and learning activity

Intervention number 1–16 (9 = control)	1–16	1	2	3	4	5	6	7	8	9	10	11	12	13	14	15	16
*n*	2529	162	175	180	159	151	167	206	154	141	122	154	163	141	157	158	139
Age (median)	45	43	38	46	44	48	43	44	47	48	48	48	47	48	43	46	44
Female gender (%)	57	60	51	65	32	62	70	73	64	40	43	37	59	63	63	65	50
BMI (median)	24.8	24.8	23.7	24.0	25.4	24.5	24.3	25.0	25.7	25.2	25.1	25.1	24.5	24.8	25.2	25.6	25.1
Swedish mother tongue (%)	86	85	83	90	88	87	87	84	85	86	84	92	93	75	85	90	86
Educational level (%)																	
Elementary school (%)	9	5	18	7	7	8	3	4	10	21	7	14	6	20	6	6	4
High school (%)	49	43	61	46	39	54	30	50	33	54	55	52	48	46	48	65	58
College/university (%)	42	52	21	47	54	38	67	46	58	26	38	34	46	34	45	29	38
Occupation at training (%)																	
Blue collar (%)	40	44	24	26	19	50	25	45	46	59	42	42	23	57	32	70	50
White collar (%)	39	48	40	39	39	38	40	51	21	37	39	36	45	40	43	27	43
Both (%)	20	8	36	36	42	12	36	4	32	4	19	22	31	3	24	3	6
No previous CPR training at all (%)	36	40	40	31	31	32	37	31	37	54	34	43	39	32	38	25	30
No previous AED training at all (%)	80	76	78	73	70	87	82	76	85	74	85	82	85	76	85	79	83
Experienced SCA (%)	8	9	5	6	10	7	7	5	11	9	13	8	7	8	5	12	6
Learning activity																	
Self-learning	1354	x	x	x	x	x	x	x	x	-	-	-	-	-	-	-	-
Mobile App instructions	676	x	x	x	x	-	-	-	-	-	-	-	-	-	-	-	-
Web-based education	1236	-	x	-	x	-	x	-	x	-	x	-	x	-	x	-	x
Reflective questions	1313	-	-	x	x	-	-	x	x	-	-	x	x			x	x
Film-based instructions	1853	-	-	-	-	x	x	x	x	x	x	x	x	x	x	x	x
Instructor-led learning	1175	-	-	-	-	-	-	-	-	x	x	x	x	x	x	x	x
Feedback device	595	-	-	-	-	-	-	-	-	-	-	-	-	x	x	x	x
Approximate time in minutes for the learning materials		030–060	060–090	045–075	075–105	060–090	090–120	075–105	105–135	075–105	105–135	090–120	120–150	090–120	130–160	105–135	135–165

*BLS*, basic life support; *CPR*, cardiopulmonary resuscitation; *AED*, automated external defibrillation; *SCA*, sudden cardiac arrest; *1–16*, BLS intervention group number 1–16; *9*, control group; *BMI*, body mass index

One or two instructors from the workplaces facilitated the instructor-led interventions. In total, 16 instructors, accredited by the Swedish resuscitation council, with about 10 years of experience were updated to 2010 ERC guidelines.

### Data Collection and Assessment

Data were collected directly after the training and 6 months after the training through individual questionnaires (Supplementary file 3) and an assessment of BLS skills in a practical test. The test was performed on a Resusci Anne full-body manikin and a HeartStart 1 AED trainer (Laerdal Medical AS, Stavanger, Norway). To compare learning outcomes, BLS skills were measured by the PC Skill Reporting system software V.2.4 (Laerdal Medical, Stavanger, Norway) and the total score (19–70 points) was calculated from the variables in the BLS algorithm using the validated modified version of the Cardiff Test of basic life support and external defibrillation (Cardiff Test, Supplementary file 4). The test was conducted as an OHCA scenario in a private setting in a converted motor home stationed outside the workplace. After permission from the participant, a stopwatch and the software automatically measured the variables and a mounted Sony HD video camera filmed the scenario. The test lasted for about 5 min. Three minutes was required for the participant to be able to recognise the OHCA victim, shout for help, call 112, ask for an AED and perform CPR, while about 2 min was required to use the AED. At 3 min from the start of the scenario, the assessor placed the AED next to the manikin. The equipment was calibrated automatically and checked manually every day, and, in the event of technical problems, an identical training manikin and AED was prepared.

### Outcome

The primary outcome was the total score for practical BLS skills calculated with the modified Cardiff Test, 6 months after training, while the secondary outcome was the total score calculated directly after training.

### Statistical Analysis

The data on participant characteristics are presented as the median or percentages.

Mixed linear regression models were applied for comparisons of the modified Cardiff Test total score to account for a potential cluster effect in the intervention groups. Due to imbalances between the groups in terms of participant characteristics, comparisons were made with adjustments for the possible confounding influence of age, gender, body mass index, mother tongue, educational level and occupation at training and previous CPR training and training on AED use.

Least square means with corresponding standard errors are presented for each intervention group, together with 95% confidence intervals and *p* values for the difference from group 9 (standard), adjusted for multiple comparisons using Dunnett’s method. Standardised effect size (Cohen’s *d*) is also given for each comparison with group 9 regarding Cardiff Test total score.

All tests are two-sided and *p* values below 0.05 were considered statistically significant. SAS for Windows version 9.4 was used for all the performed analyses.

### Ethics

The Regional Ethical Review Board in Gothenburg approved the ethical application on 23 March 2014 (2014/134-14).

## Results

**Fig. 1:**
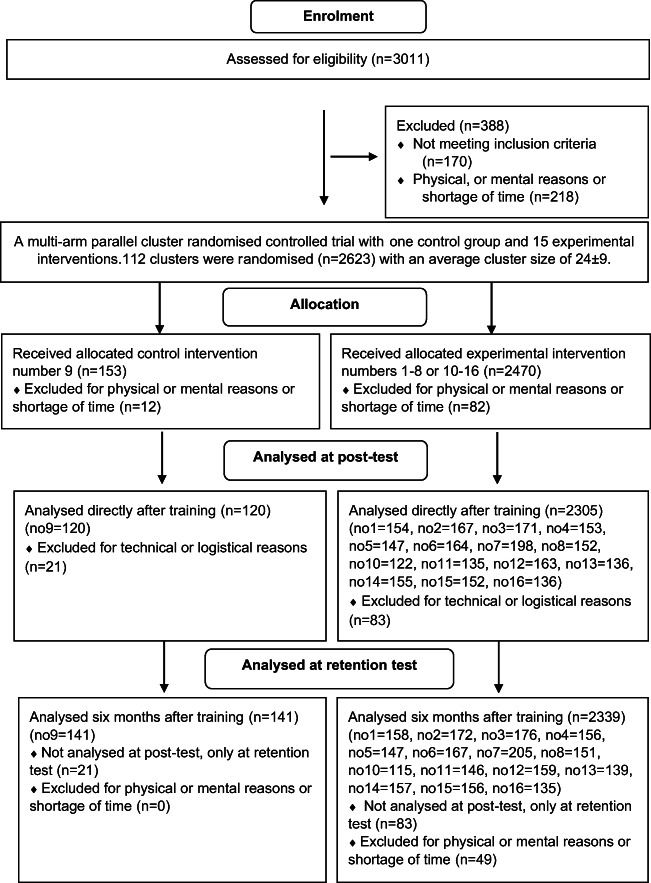
CONSORT flow diagram. 15 different basic life support (BLS) training interventions were compared in a multi-arm parallel design with one control group, number 9 (no. 9), i.e. a standard instructor-led and film-based BLS training. The experimental interventions from the multi-arm cluster randomised controlled trial, numbers 1–8 and 10–16, were additional to practical BLS training and consisted of seven learning activities in different combinations. The total number of participants for each intervention, 1–16, is shown in parentheses

This multi-arm parallel cluster RCT enrolled 2623 laypersons recruited from workplaces in a BLS educational project from 2014 to 2016, of which 2529 were eligible for analysis either at 6 months (*n* = 2480) or directly (*n* = 2425) after training. The characteristics of the participants are presented in Table 1. The participants were randomised to one of 16 adult BLS training interventions with different additional learning activities (Fig. 1). For physical and mental reasons and because of a shortage of time and stress, 94 participants were lost to follow-up directly after training and 49 between the tests. For the primary outcome, a total score for practical skills calculated with the modified Cardiff Test 6 months after training, 2480 individuals were analysed. For the secondary outcome, 2425 individuals were analysed as 104 individual tests were incomplete for analysis at the test directly after training but were complete for analysis at the test 6 months after training. The mean total score for the modified Cardiff Test (19–70 points) including all 16 groups was 59.1 (84%) directly after training and 58.4 (83%) 6 months after training.

### Primary Outcome

Due to imbalances between the intervention groups in terms of participant characteristics, comparisons were adjusted for these imbalances.

For the primary outcome, i.e. BLS practical skills 6 months after training, four of the interventions obtained a significantly higher modified Cardiff Test total score compared with the control group (number 9), i.e. BLS instructor-led practical training with film-based instructions (Table 2). The standardised effect size is also presented for differences from the control group 9 in Table 2. The four interventions are as follows:

1. BLS training intervention number 16, including all additional learning activities, i.e. a preparatory web-based education, instructor-led training, film-based instructions, three reflective essential questions and use of a chest compression feedback device
2. BLS training intervention number 11, with instructor-led training including film-based instructions and three reflective questions
3. BLS training intervention number 10, including a preparatory web-based education, instructor-led training and film-based instructions
4. BLS training intervention number 14, including a preparatory web-based education, instructor-led training, film-based instructions and compression feedback.

**Table 2:** Total score practical skills in BLS, 6 months after training

	Unadjusted				Adjusted^#^			
	lsmeans ± stderr*	95% CI**	p**	Effect size‘**’	lsmeans ± stderr*	95% CI**	p**	Effect size‘**’
Intervention								
1	57.0 ± 0.53	− 1.4 to 3.0	0.95	0.16	55.7 ± 0.51	− 2.1 to 1.9	1.00	− 0.03
2	58.0 ± 0.54	− 0.5 to 4.0	0.20	0.24	56.9 ± 0.51	− 1.1 to 3.0	0.77	0.19
3	58.3 ± 0.54	− 0.1 to 4.3	0.08	0.39	57.1 ± 0.51	− 0.8 to 3.3	0.50	0.23
4	58.9 ± 0.55	0.5 to 5.0	0.008	0.53	57.6 ± 0.52	− 0.3 to 3.8	0.15	0.34
5	57.5 ± 0.53	− 0.9 to 3.5	0.52	0.25	56.8 ± 0.52	− 1.1 to 2.9	0.85	0.17
6	57.8 ± 0.53	− 0.6 to 3.8	0.29	0.31	56.4 ± 0.51	− 1.5 to 2.6	1.00	0.11
7	58.2 ± 0.49	− 0.1 to 4.2	0.07	0.38	57.1 ± 0.47	− 0.7 to 3.2	0.40	0.24
8	58.8 ± 0.56	0.4 to 4.9	0.01	0.50	57.9 ± 0.54	− 0.1 to 4.1	0.07	0.38
10	59.0 ± 0.62	0.4 to 5.2	0.01	0.51	58.1 ± 0.58	0.1 to 4.4	0.03	0.42
11	59.0 ± 0.57	0.5 to 5.1	0.008	0.51	58.5 ± 0.55	0.5 to 4.7	0.006	0.48
12	58.4 ± 0.53	− 0.0 to 4.4	0.05	0.40	57.4 ± 0.51	− 0.5 to 3.5	0.24	0.28
13	58.1 ± 0.54	− 0.3 to 4.2	0.14	0.34	57.6 ± 0.52	− 0.3 to 3.7	0.16	0.31
14	59.2 ± 0.54	0.7 to 5.2	0.003	0.58	58.1 ± 0.52	0.2 to 4.3	0.02	0.44
15	58.5 ± 0.49	0.2 to 4.5	0.03	0.44	57.5 ± 0.50	− 0.3 to 3.6	0.17	0.31
16	60.0 ± 0.55	1.5 to 6.0	< 0.0001	0.72	58.8 ± 0.54	0.9 to 5.0	0.001	0.56
9 control	56.2 ± 0.57	-	-	-	55.9 ± 0.54	-	-	-

^#^Adjusted for age, mother tongue, educational level, previous cardiopulmonary resuscitation (CPR) training, gender, body mass index (BMI), occupation and previous automated external defibrillation (AED) use training. *Least square means with corresponding standard errors. *CI*, confidence interval; *p*, *p* values below 0.05 were considered statistically significant. **For comparison with the control group. ‘**’Standardised effect size (Cohen’s *d*) for comparison with control group 9 (all interventions included)

A description of individual items in the Cardiff Test score (Table 3) and separate variables (Table 4) for these four interventions and the control group are given as unadjusted comparisons. Although all but one of the other eleven training interventions obtained a higher total score than the control group, none of these differences was statistically significant.

**Table 3:** Cardiff Test, 6 months after intervention. Control group 9 versus interventions 10, 11, 14 and 16

	Control	Intervention	Intervention	Intervention	Intervention
	9	10	11	14	16
Individual items	(*n* = 141)	(*n* = 115)	(*n* = 146)	(*n* = 157)	(*n* = 135)
Checks responsiveness—by talking					
2 p. Yes	95.7	97.4	95.2	98.7	94.1
1 p. No	4.3	2.6	4.8	1.3	5.9
Checks responsiveness—by shaking					
3 p. Yes	93.6	97.4	93.8	98.7	93.3
2 p. No	5.0	2.6	5.5	1.3	6.7
1 p. Potentially dangerous	1.4	0	0.7	0	0
Opens airway—by head tilt and chin lift					
5 p. Perfect as instructed	24.8	43.5	41.8	42.0	40.0
4 p. Acceptable	9.9	6.1	11.0	8.3	9.6
3 p. Attempted other	2.8	0.9	2.1	0.6	0
2 p. Attempted visible but fails	14.9	17.4	15.8	29.9	24.4
1 p. No	47.5	32.2	29.5	19.1	25.9
Checks breathing—by look, listen and feel					
2 p. Yes	75.2	80.0	85.6	87.3	87.4
1 p. No	24.8	20.0	14.4	12.7	12.6
Calls 112 or shouts for call to 112					
2 p. Yes	90.1	88.7	95.9	98.7	97.8
1 p. No	9.9	11.3	4.1	1.3	2.2
Asks for AED					
2 p. Yes	77.3	89.6	87.0	84.1	92.6
1 p. No	22.7	10.4	13.0	15.9	7.4
Starts CPR—compression/ventilations ratio					
4 p. 30:2 (28–32:2)	63.1	77.4	74.7	86.0	79.3
3 p. Another ratio	35.5	11.7	25.3	14.0	20.0
2 p. Compressions only	1.4	0.9	0	0	0.7
1 p. Ventilations only	0	0	0	0	0
Hand placement compressions					
4 p. Correct	18.4	29.6	46.6	13.4	22.2
3 p. Other wrong	39.7	41.7	36.3	44.6	52.6
2 p. Too low	41.8	28.7	17.1	42.0	25.2
1 p. Not attempted	0	0	0	0	0
Average compression depth					
6 p. 50–59 mm	59.6	60.0	62.3	60.5	63.7
5 p. ≥ 60 mm	12.8	13.9	6.8	23.6	18.5
4 p. 35–49 mm	24.8	23.5	28.8	14.6	15.6
2 p. < 35 mm	2.8	2.6	2.1	1.3	2.2
1 p. Not attempted	0	0	0	0	0
Average compression rate					
6 p. 100–120	36.2	35.7	45.2	47.1	46.7
5 p. 121–140	7.1	13.9	14.4	13.4	18.5
4 p. 80–99	33.3	34.8	23.3	26.1	21.5
3 p. > 140	0.7	1.7	1.4	1.3	0.7
2 p. < 80	22.7	13.9	15.8	12.1	12.6
1 p. Not attempted	0	0	0	0	0
Total compressions counted					
6 p. 140–190	56.0	62.6	64.4	50.3	59.3
5 p. > 190	16.3	13.0	11.6	22.3	18.5
4 p. 121–139	12.1	9.6	8.2	10.2	10.4
3 p. 81–120	12.1	10.4	13.0	15.3	10.4
2 p. ≤ 80	3.5	4.3	2.7	1.9	1.5
1 p. Not attempted	0	0	0	0	0
Average ventilation volume					
5 p. 500–600 ml	7.8	5.2	15.1	11.5	17.0
4 p. 1–499 ml	14.2	8.7	13.7	7.6	9.6
` 3 p. > 600 ml	55.3	79.1	63.0	70.7	63.7
2 p. 0 ml	21.3	6.1	8.2	10.2	8.9
1 p. Not attempted	1.4	0.9	0	0	0.7
Total ventilations counted					
5 p. 8–12	43.3	62.6	60.3	60.5	60.0
4 p. 1–7	20.6	20.0	18.5	16.6	18.5
3 p. > 12	13.5	10.4	13.0	12.7	11.9
2 p. 0	21.3	6.1	8.2	10.2	8.9
1 p. Not attempted	1.4	0.9	0	0	0.7
Total hands-off time					
4 p. ≤ 60 s	4.3	3.5	4.1	7.6	4.4
3 p. 61–90 s	61.0	61.7	58.2	68.2	64.4
2 p. 91–135 s	32.6	32.2	35.6	24.7	30.4
1 p. > 135 s	2.1	2.6	2.1	0.6	0.7
Switches on AED					
2 p. Yes	100	100	100	99.4	99.3
1 p. No	0	0	0	0.6	0.7
Attaches electrode pads					
6 p. Both pads completely in areas	71.6	95.7	85.6	90.4	97.0
5 p. One in area, one crossing border of area	12.1	1.7	5.5	2.5	0.7
4 p. One in area, one outside area	10.6	1.7	3.4	3.8	0.7
3 p. Both crossing border of area	2.8	0.9	2.1	0	0.7
2 p. Both outside areas	2.8	0	3.4	2.5	0
1 p. Not attached or not plugged into AED	0	0	0	0.6	0.7
Checks safety, ensures nobody in contact with manikin					
2 p. Yes	42.6	65.2	64.4	53.5	65.2
1 p. No	57.4	34.8	35.6	46.5	34.8
Delivers shock as directed by AED					
2 p. Yes	100	99.1	100	99.4	99.3
1 p. No	0	0.9	0	0.6	0.7
Resumes CPR immediately after shock					
2 p. Yes	87.2	91.3	89.7	89.2	88.9
1 p. No	12.8	8.7	10.3	10.8	11.1

*Cardiff Test*, Cardiff Test of basic life support (BLS) and automated external defibrillation (AED); *CPR*, cardiopulmonary resuscitation. Data was collected from the Resusci Anne Manikin connected to the PC Skill Reporting system (Laerdal Medical, Stavanger, Norway) and from direct observation. All results are crude proportions (%), i.e. not adjusted for clustering or imbalances in participants’ characteristics. All available data were used

**Table 4:** Separate variables, 6 months after intervention. Control group 9 versus interventions 10, 11, 14 and 16

Variables	Control	Intervention	Intervention	Intervention	Intervention
	9	10	11	14	16
Correct compressions (%)	*N* = 141	*N* = 115	*N* = 146	*N* = 157	*N* = 135
Median	29	48	49	48	63
25th, 75th percentile	2, 74	4, 84	18, 85	8, 79	14, 92
Compressions with insufficient depth (%)	*N* = 141	*N* = 115	*N* = 146	*N* = 157	*N* = 135
Median	5	6	13	3	2
25th, 75th percentile	1, 47	1, 38	2, 51	1, 21	1, 23
Compressions with incorrect hand-position (%)	*N* = 141	*N* = 115	*N* = 146	*N* = 157	*N* = 135
Median	42	23	3	11	18
25th, 75th percentile	6, 84	0, 65	0, 49	0, 41	0, 69
Compressions with incomplete release (%)	*N* = 141	*N* = 115	*N* = 146	*N* = 157	*N* = 135
Median	0	0	0	0	0
25th, 75th percentile	0, 1	0, 1	0, 0	0, 0	0, 0
> 0 (%)	26.2	30.4	24.0	24.8	21.5
Average compression depth (mm)	*N* = 141	*N* = 115	*N* = 146	*N* = 157	*N* = 135
Median	54	55	54	57	57
25th, 75th percentile	47, 59	48, 58	48, 58	53, 59	53, 59
Average compression rate (per minute)	*N* = 141	*N* = 115	*N* = 146	*N* = 157	*N* = 135
Median	96	100	103	105	109
25th, 75th percentile	81, 109	86, 115	86, 113	91, 116	89, 117
Correct ventilations (%)	*N* = 141	*N* = 115	*N* = 146	*N* = 157	*N* = 135
Median	1	1	1	1	0
25th, 75th percentile	0, 3	0, 2	0, 3	0, 3	0, 3
> 0 (%)	53.2	53.9	63.0	63.1	50.4
Average ventilation volume (ml)	*N* = 141	*N* = 115	*N* = 146	*N* = 157	*N* = 135
Median	662	900	731	795	820
25th, 75th percentile	365, 1000	651, 1271	522, 1070	547, 1073	546, 1226
Time to start of CPR (seconds)	*N* = 141	*N* = 115	*N* = 146	*N* = 157	*N* = 135
Median	27	28	29	25	29
25th, 75th percentile	19, 35	23, 38	22, 36	19, 34	20, 35
Time to 1st shock (seconds)	*N* = 141	*N* = 115	*N* = 145	*N* = 156	*N* = 134
Median	74	67	71	66	66
25th, 75th percentile	64, 86	59, 78	59, 84	57, 78	58, 76

Separate variables, variables for quality of practical skills for cardiopulmonary resuscitation (CPR) and automated external defibrillation (AED), 6 months after training in basic life support (BLS)

Data was collected from the Resusci Anne Manikin connected to the PC Skill Reporting system (Laerdal Medical, Stavanger, Norway) and from direct observation. All results are crude, i.e. not adjusted for clustering or imbalances in participants’ characteristics. All available data was used

### Secondary Outcome

For secondary outcome, i.e. BLS skills directly after training, intervention numbers 16, 12 and 14, all including a preparatory web-based interactive education, instructor-led training and film-based instructions, obtained a significantly higher modified Cardiff Test total score compared with the control group, when adjustment was made for imbalances in participants’ characteristics (Table 5).

**Table 5:** Total score practical skills in BLS, directly after training

	Unadjusted				Adjusted^#^			
	lsmeans ± stderr*	95% CI**	p**	Effect size‘**’	lsmeans ± stderr*	95% CI**	p**	Effect size‘**’
Intervention								
1	56.8 ± 0.57	− 3.9 to 0.9	0.49	− 0.27	56.0 ± 0.55	− 4.2 to 0.3	0.12	− 0.35
2	57.5 ± 0.59	− 3.2 to 1.7	0.99	− 0.15	56.6 ± 0.56	− 3.7 to 0.9	0.47	− 0.28
3	58.3 ± 0.59	− 2.5 to 2.5	1.00	− 0.00	57.4 ± 0.56	− 2.9 to 1.7	1.00	− 0.12
4	58.5 ± 0.59	− 2.2 to 2.7	1.00	0.05	57.4 ± 0.56	− 2.9 to 1.7	1.00	− 0.13
5	57.3 ± 0.56	− 3.4 to 1.4	0.88	− 0.21	56.7 ± 0.55	− 3.5 to 0.9	0.57	− 0.26
6	58.5 ± 0.58	− 2.2 to 2.7	1.00	0.05	57.5 ± 0.56	− 2.8 to 1.8	1.00	− 0.10
7	59.3 ± 0.54	− 1.4 to 3.3	0.90	0.21	58.5 ± 0.53	− 1.7 to 2.7	1.00	0.11
8	58.5 ± 0.60	− 2.2 to 2.8	1.00	0.05	57.9 ± 0.58	− 2.4 to 2.3	1.00	− 0.01
10	60.8 ± 0.67	− 0.2 to 5.1	0.08	0.56	60.0 ± 0.63	− 0.4 to 4.4	0.17	0.45
11	59.8 ± 0.61	− 1.0 to 4.0	0.54	0.32	59.2 ± 0.59	− 1.1 to 3.6	0.66	0.27
12	61.4 ± 0.56	0.7 to 5.5	0.005	0.74	60.5 ± 0.54	0.3 to 4.8	0.02	0.61
13	59.4 ± 0.58	− 1.3 to 3.6	0.81	0.25	59.1 ± 0.56	− 1.2 to 3.3	0.78	0.24
14	61.3 ± 0.59	0.6 to 5.5	0.007	0.68	60.5 ± 0.57	0.2 to 4.8	0.02	0.56
15	60.0 ± 0.52	− 0.6 to 4.1	0.26	0.40	59.3 ± 0.53	− 0.9 to 3.5	0.51	0.30
16	61.6 ± 0.58	0.9 to 5.8	0.002	0.78	60.8 ± 0.57	0.5 to 5.1	0.008	0.65
9 control	58.3 ± 0.64		-	-	58.0 ± 0.61	-	-	-

^#^Adjusted for age, mother tongue, educational level, previous cardiopulmonary resuscitation (CPR) training, gender, body mass index (BMI), occupation and previous automated external defibrillation (AED) use training. *Least square means with corresponding standard errors. *CI*, confidence interval; *p*, *p* values below 0.05 were considered statistically significant. **For comparison with the control group. ‘**’Standardised effect size (Cohen’s *d*) for comparison with control group 9 (all interventions included)

Theoretical knowledge on first action if stroke or AMI and OHCA, i.e. call 112, general symptoms of stroke and AMI and healthy lifestyle factors, self-assessed theoretical knowledge and practical skills, confidence and willingness to act in a real-life OHCA situation, directly and 6 months after training are described in Supplementary file 5, Table 6a and 6b respectively.

## Discussion

The main results in this cluster RCT showed that a preparatory interactive web-based education on CVD, a feedback device on the quality of compressions and reflective essential questions, in addition to instructor-led and film-based training, appear to provide potential benefits for practical skills in BLS when compared with the standard, 6 months after training. However, the clinical impact and the way these three additional learning activities could be combined in an optimal way have been investigated but it is still unclear and further studies are required to explore this issue.

Yet, all three learning activities have previously been reported to be beneficial for learning. Firstly, E-learning has been reported to be as good as lectures and teaching before training [2, 37–39], and a web-based education prior to practical training in BLS to laypersons showed a slightly higher total score compared to BLS training without the web-based education [31]. Secondly, CPR feedback devices have resulted in improved quality in CPR and are recommended in training [2, 6]. Thirdly, some form of reflection is proven to increase effective learning in an educational setting [8, 40].

Moreover, the results in this study indicate that an instructor who facilitated the interventions appeared to be favourable for learning. This is in line with a systematic review by Chen et al., who presented interventions that improved high-quality CPR by laypersons and the result showed that an instructor or dispatcher was engaged in most of the studies which improved efficacy [17], even if self-instruction is recommended as an alternative [2, 4]. The instructor-led training in this study offered some communication with both the instructor and the other participants. For example, in the short scenario with the small manikin at the end of the training, the participants collaborated in pairs in a brief OHCA scenario and communication may be a positive factor for learning. Experience, reflection, understanding and hands-on training may all increase learning, according to Kolb [8], may be extended by peer learning and may come closer to experiential learning [4, 8]. Reflection on essential questions was included in half the interventions and was discussed in pairs and some peer learning may have occurred, but we are unable to confirm this. The self-learning participants reflected on their own and missed the social collaboration in the group. Reflection individually has been criticised in educational settings for the lack of socio-cultural awareness and opportunity for sense-making [41]. In contrast, one study has shown non-inferiority compared with instructor-led BLS training [42] and there are opportunities for self-directed learning if they can be combined with, for example, workplace collaboration and collaborative technological platforms [43].

Furthermore, compression-only CPR, real-time feedback and mobile devices with CPR instructions are factors which appear to improve efficacy [17]. Moreover, eight of the interventions were self-directed and none of them was in favour of the control group. A mobile application gave the CPR instructions for learning in four of the interventions, and even if mobile devices may delay the early start of CPR [17], this is an easy way to learn by yourself. On the other hand, the instruction in the mobile application was only 30 min and much shorter than that in the other interventions but we did not estimate how long time the participants actually trained the BLS tasks.

In addition to the main result, two of the learning activities, numbers 16 and 14, resulted in improvement both directly after training and 6 months after training. Six months can be regarded as long-term retention as practical skills deteriorate after 3 to 6 months [2, 6], but, for a workplace organisation with laypersons, it may be difficult to organise retraining every 6 months. In an RCT in 2015, Nishiyama et al. showed that a 15-min simple chest compression-only BLS retraining 6 months after training resulted in retained efficacy in calling the EMS and chest compression skills up to 1 year [16] and this may be appropriate to use in a workplace environment.

In this study and according to the national guidelines in BLS, the instructor gave verbal feedback and helped the participants visually to achieve the learning objective and the participants self-assessed their practical skills with the manikin. Self-assessment is possible in both instructor-directed and self-directed training, but, if the assessment of the participants was performed in communication with the instructor and using technological feedback, we might have another result. Assessment is recommended for long-term retention with mastery learning [2, 6]. Unpredictably, directly after training, the control group, i.e. the standard group, showed a poorer result than half the interventions but without any significant difference. Learning activities without formal assessment related to the learning goal might be a weakness for learning in BLS, especially without objective and technological support [20, 44–46]. The small training manikin and the intervention itself might therefore be a weakness for the learning outcome.

Additionally, when comparing interventions numbers 10, 11, 14 and 16 with the control group 9, several individual items in the Cardiff Test and separate variables obtained higher scores.

Consequently, the preparatory interactive web-based education in addition to the instructor-led and film-based training was involved when improvement compared with standard was found at primary outcome 6 months after training. Adding theoretical knowledge on CVD as stroke, AMI and OHCA, and BLS, CPR and AED and healthy lifestyle factors may provide motivation for learning practical skills, but this is unclear. For further implementation, the web-based education could also include some synchronous brief practical self-retraining with a simple manikin, with feedback and encouragement to collaborate at the workplace and be spaced over the year. This might increase the level of re-qualified potential bystanders, as well as being easy to update, and it may reduce the financial costs and organisational affordances [11]. The educational framework at workplaces includes different types of learners and therefore needs various effective blended and spaced learning activities to enable improved learning in BLS, but we did not account for that in the study. This may be the subject of a further study.

With this third study, we conclude that the learning activities of a preparatory interactive web-based education on stroke, AMI, OHCA, CPR, AED and healthy lifestyle factors, an instructor present, film-based instructions, reflective questions and chest compressions feedback may have benefits for learning in BLS compared with instructor-led and film-based instructions alone.

## Strengths and Limitations

The cluster randomised design targeted at a study population at workplaces was a strength as was the large sample size. Limitations include, in spite of the randomisation design, imbalances between the intervention groups regarding baseline characteristics. In addition, since we have no exact data on the actual time spent on each intervention, we could not adjust for this factor, which is a limitation. Thus, we cannot exclude the possibility that some of the differences that were found between groups may have been explained by a different duration of the training. The duration of the self-learning training was not exactly defined and that is a limitation of the study design. Furthermore, it might be argued whether the statistically significant differences are of clinical relevance since the effect size was small to moderate.

## Conclusion

BLS practical training, adding different combinations of a preparatory web-based interactive education, reflective questions and chest compression feedback to instructor-led training and film-based instructions, obtained higher modified Cardiff Test total scores 6 months after training compared with instructor-led and film-based BLS training alone. The differences were small in magnitude and the clinical relevance of our findings needs to be further explored.

## Supplementary Information

ESM 1 (DOCX 36 kb)

ESM 2 (DOCX 21 kb)

ESM 3 (DOCX 62 kb)

ESM 4 (DOCX 61 kb)

ESM 5 (DOCX 21.6 kb)

## Data Availability

All data generated and analysed during this study are included in this published article and its supplementary information files.

## Abbreviations

AED: automated external defibrillation
AMI: acute myocardial infarction
BLS: basic life support
CI: confidence interval
CPR: cardiopulmonary resuscitation
CVD: cardiovascular disease
EMS: emergency medical service
ERC: European Resuscitation Council
OHCA: out-of-hospital cardiac arrest
PROBE: Prospective Randomised Open Blinded End-Point Evaluation
RCT: randomised, controlled trial
SCA: sudden cardiac arrest
SRC: Swedish Resuscitation Council
