# Supplementary material for: Learning Outcome After Different Combinations of Seven Learning Activities in Basic Life Support on Laypersons in Workplaces: a Cluster Randomised, Controlled Trial
Source: Med Sci Educ. 2020 Nov 18;31(1):161–73. doi: 10.1007/s40670-020-01160-3 (PMC8368380; doi:10.1007/s40670-020-01160-3)
Supplement: Supplementary file 1 — (DOCX 36 kb) [file 40670_2020_1160_MOESM1_ESM.docx]

**Supplementary file 1, Table 1 and Table 2**
The Journal Medical Science Educator. Title: Learning outcome after different combinations of seven learning activities in basic life support on laypersons in workplaces: a cluster randomised, controlled trial, by Bylow et al. Corresponding author: Helene Bylow [helene.bylow@gu.se](mailto:helene.bylow@gu.se)

**Table 1: CONSORT 2010 checklist of information to include when reporting a cluster randomised trial**

| Section/Topic | Item No | Standard Checklist item | Extension for cluster designs | Page No * |
| --- | --- | --- | --- | --- |
| Title and abstract | | | |  |
|  | 1a | Identification as a randomised trial in the title | Identification as a cluster randomised trial in the title | Page 1 |
|  | 1b | Structured summary of trial design, methods, results, and conclusions (for specific guidance see CONSORT for abstracts)^[[1]](#endnote-1),^^[[2]](#endnote-2)^ | See table 2 | Page 2 |
| Introduction | | | |  |
| Background and objectives | 2a | Scientific background and explanation of rationale | Rationale for using a cluster design | Page 2, Abstract section. Page 5 Background section. Page 5, Methods section |
|  | 2b | Specific objectives or hypotheses | Whether objectives pertain to the cluster level, the individual participant level or both | Page 6,7,8,9, Methods section. |
| Methods | | | |  |
| Trial design | 3a | Description of trial design (such as parallel, factorial) including allocation ratio | Definition of cluster and description of how the design features apply to the clusters | Page 5, 6, Methods section. Study design paragraph. |
|  | 3b | Important changes to methods after trial commencement (such as eligibility criteria), with reasons |  | Not applicable |
| Participants | 4a | Eligibility criteria for participants | Eligibility criteria for clusters | Page 6, Methods section. Page 10, Results section. |
|  | 4b | Settings and locations where the data were collected |  | Page 8, Methods section. Page 10, Results section. |
| Interventions | 5 | The interventions for each group with sufficient details to allow replication, including how and when they were actually administered | Whether interventions pertain to the cluster level, the individual participant level or both | Page 6, 9, Methods section. Statistical analysis paragraph. |
| Outcomes | 6a | Completely defined pre-specified primary and secondary outcome measures, including how and when they were assessed | Whether outcome measures pertain to the cluster level, the individual participant level or both | Page 9, Methods section. Statistical analysis paragraph. |
|  | 6b | Any changes to trial outcomes after the trial commenced, with reasons |  | Not applicable |
| Sample size | 7a | How sample size was determined | Method of calculation, number of clusters(s) (and whether equal or unequal cluster sizes are assumed), cluster size, a coefficient of intracluster correlation (ICC or *k*), and an indication of its uncertainty | Page 6, Methods section. |
|  | 7b | When applicable, explanation of any interim analyses and stopping guidelines |  | Not applicable |
| Randomisation: | | | |  |
| Sequence generation | 8a | Method used to generate the random allocation sequence |  | Page 6, Methods section. |
|  | 8b | Type of randomisation; details of any restriction (such as blocking and block size) | Details of stratification or matching if used | Page 6, Methods section. |
| Allocation concealment mechanism | 9 | Mechanism used to implement the random allocation sequence (such as sequentially numbered containers), describing any steps taken to conceal the sequence until interventions were assigned | Specification that allocation was based on clusters rather than individuals and whether allocation concealment (if any) was at the cluster level, the individual participant level or both | Page 6, Methods section. |
| Implementation | 10 | Who generated the random allocation sequence, who enrolled participants, and who assigned participants to interventions | Replace by 10a, 10b and 10c | Page 5, 6, 7, 8, Methods section. |
|  | 10a |  | Who generated the random allocation sequence, who enrolled clusters, and who assigned clusters to interventions | Page 5, 6, 7, 8, Methods section. |
|  | 10b |  | Mechanism by which individual participants were included in clusters for the purposes of the trial (such as complete enumeration, random sampling) | Page 5, 6, 7, 8, Methods section. |
|  | 10c |  | From whom consent was sought (representatives of the cluster, or individual cluster members, or both), and whether consent was sought before or after randomisation | Page 7, Methods section. |
|  |  |  |  |  |
| Blinding | 11a | If done, who was blinded after assignment to interventions (for example, participants, care providers, those assessing outcomes) and how |  | Page 6, Methods section. |
|  | 11b | If relevant, description of the similarity of interventions |  | Not applicable |
| Statistical methods | 12a | Statistical methods used to compare groups for primary and secondary outcomes | How clustering was taken into account | Page 9, Methods section. |
|  | 12b | Methods for additional analyses, such as subgroup analyses and adjusted analyses |  | Page 9, Methods section. |
| Results | | | |  |
| Participant flow (a diagram is strongly recommended) | 13a | For each group, the numbers of participants who were randomly assigned, received intended treatment, and were analysed for the primary outcome | For each group, the numbers of clusters that were randomly assigned, received intended treatment, and were analysed for the primary outcome | Page 10, Results section. CONSORT Flow Diagram Fig. 1 |
|  | 13b | For each group, losses and exclusions after randomisation, together with reasons | For each group, losses and exclusions for both clusters and individual cluster members | Page 10, Results section. CONSORT Flow Diagram Fig. 1 |
| Recruitment | 14a | Dates defining the periods of recruitment and follow-up |  | Page 5, Methods section. Page 10, Results section. |
|  | 14b | Why the trial ended or was stopped |  | Not applicable |
| Baseline data | 15 | A table showing baseline demographic and clinical characteristics for each group | Baseline characteristics for the individual and cluster levels as applicable for each group | Table 1 |
| Numbers analysed | 16 | For each group, number of participants (denominator) included in each analysis and whether the analysis was by original assigned groups | For each group, number of clusters included in each analysis | Page 10, Results section. CONSORT Flow Diagram Fig. 1 |
| Outcomes and estimation | 17a | For each primary and secondary outcome, results for each group, and the estimated effect size and its precision (such as 95% confidence interval) | Results at the individual or cluster level as applicable and a coefficient of intracluster correlation (ICC or k) for each primary outcome | Table 2, 3, 4, 5 |
|  | 17b | For binary outcomes, presentation of both absolute and relative effect sizes is recommended |  | Not applicable |
| Ancillary analyses | 18 | Results of any other analyses performed, including subgroup analyses and adjusted analyses, distinguishing pre-specified from exploratory |  | Table 2, 3, 4, 5 |
| Harms | 19 | All important harms or unintended effects in each group (for specific guidance see CONSORT for harms^[[3]](#endnote-3)^) |  | Not applicable |
| Discussion | | | |  |
| Limitations | 20 | Trial limitations, addressing sources of potential bias, imprecision, and, if relevant, multiplicity of analyses |  | Not applicable |
| Generalisability | 21 | Generalisability (external validity, applicability) of the trial findings | Generalisability to clusters and/or individual participants (as relevant) | Page 10, Results section. |
| Interpretation | 22 | Interpretation consistent with results, balancing benefits and harms, and considering other relevant evidence |  | Page 10, Results section. |
| Other information | | |  |  |
| Registration | 23 | Registration number and name of trial registry |  | Page 3, Abstract section. Page 6, Methods section.  ClinicalTrials.gov (ID: NCT03618888). |
| Protocol | 24 | Where the full trial protocol can be accessed, if available |  | Page 3, Abstract section. Page 6, Methods section.  ClinicalTrials.gov (ID: NCT03618888). |
| Funding | 25 | Sources of funding and other support (such as supply of drugs), role of funders |  | Page 19, 20, Declarations section |

** Note: page numbers optional depending on journal requirements*

**Additional file 1**
Learning outcome after different combinations of seven learning activities in basic life support on laypersons in workplaces: a hypothesis generating cluster randomised, controlled trial, by Bylow et al. March 19, 2020

**Table 2: Extension of CONSORT for abstracts**1**^,^**2 **to reports of cluster randomised trials**

| Item | Standard Checklist item | Extension for cluster trials |
| --- | --- | --- |
| Title | Identification of study as randomised | Identification of study as cluster randomised |
| Trial design | Description of the trial design (e.g. parallel, cluster, non-inferiority) | Page 1, Title section. Page 2, Abstract section. Page 6, Methods section. |
| Methods |  |  |
| Participants | Eligibility criteria for participants and the settings where the data were collected | Eligibility criteria for clusters. Page 6, 8, Methods section. |
| Interventions | Interventions intended for each group |  |
| Objective | Specific objective or hypothesis | Whether objective or hypothesis pertains to the cluster level, the individual participant level or both. Page 2, Abstract section. Page 5, Background section. |
| Outcome | Clearly defined primary outcome for this report | Whether the primary outcome pertains to the cluster level, the individual participant level or both. Page 2, Abstract section. Page 9, Methods section. |
| Randomization | How participants were allocated to interventions | How clusters were allocated to interventions. Page 2, Abstract section. Page 6, methods section. |
| Blinding (masking) | Whether or not participants, care givers, and those assessing the outcomes were blinded to group assignment | Page 6, Methods section. |
| Results |  |  |
| Numbers randomized | Number of participants randomized to each group | Number of clusters randomized to each group. Page 6, Methods section. |
| Recruitment | Trial status^^[[4]](#footnote-1)^^ |  |
| Numbers analysed | Number of participants analysed in each group | Number of clusters analysed in each group. Page 10, Results section. CONSORT Flow Diagram Fig. 1 |
| Outcome | For the primary outcome, a result for each group and the estimated effect size and its precision | Results at the cluster or individual participant level as applicable for each primary outcome. Page 10, Results section. CONSORT Flow Diagram Fig. 1 |
| Harms | Important adverse events or side effects | Not applicable |
| Conclusions | General interpretation of the results | Page 2, Abstract section. Page 10-14, Results section |
| Trial registration | Registration number and name of trial register | Page 3, Abstract section. Page 6, Methods section. |
| Funding | Source of funding | Page 19, 20, Declarations section. |
|  |  |  |

**REFERENCES**

1. Hopewell S, Clarke M, Moher D, Wager E, Middleton P, Altman DG, et al. CONSORT for reporting randomised trials in journal and conference abstracts. *Lancet* 2008, 371:281-283 [↑](#endnote-ref-1)
2. Hopewell S, Clarke M, Moher D, Wager E, Middleton P, Altman DG at al (2008) CONSORT for reporting randomized controlled trials in journal and conference abstracts: explanation and elaboration. *PLoS Med* 5(1): e20 [↑](#endnote-ref-2)
3. Ioannidis JP, Evans SJ, Gotzsche PC, O'Neill RT, Altman DG, Schulz K, Moher D. Better reporting of harms in randomized trials: an extension of the CONSORT statement. *Ann Intern Med* 2004; 141(10):781-788. [↑](#endnote-ref-3)
4. Relevant to Conference Abstracts [↑](#footnote-ref-1)
