# Supplementary material for: Learning Outcome After Different Combinations of Seven Learning Activities in Basic Life Support on Laypersons in Workplaces: a Cluster Randomised, Controlled Trial
Source: Med Sci Educ. 2020 Nov 18;31(1):161–73. doi: 10.1007/s40670-020-01160-3 (PMC8368380; doi:10.1007/s40670-020-01160-3)
Supplement: Supplementary file 2 — (DOCX 21 kb) [file 40670_2020_1160_MOESM2_ESM.docx]

**Supplementary file 2 – The BLS training intervention, numbers 1-16**Medical Science Educator. Title: Learning outcome after different combinations of seven learning activities in basic life support on laypersons in workplaces: a cluster randomised, controlled trial, by Bylow et al. Corresponding author: Helene Bylow. [helene.bylow@gu.se](mailto:helene.bylow@gu.se)

| **The BLS training intervention, numbers 1-16** | | | |
| --- | --- | --- | --- |
| **1.**  Self-learning App-instruction | **5.** Self-learning  Film-instruction | **9. Control** Instructor-led Film-instruction | **13.** Instructor-led  Film-instruction Comp-feedback |
| **2.** Self-learning  App-instruction Web-education | **6.**  Self-learning  Film-instruction Web-education | **10.**  Instructor-led  Film-instruction Web-education | **14.** Instructor-led  Film-instruction Web-education  Comp-feedback |
| **3.** Self-learning  App-instruction Reflective-questions | **7.**  Self-learning  Film-instruction Reflective-questions | **11.**  Instructor-led  Film-instruction Reflective-questions | **15.**  Instructor-led  Film-instruction Reflective-questions Comp-feedback |
| **4.**  Self-learning  App-instruction Web-education  Reflective-questions | **8.**  Self-learning  Film-instruction Web-education  Reflective-questions | **12.**  Instructor-led  Film-instruction Web-education  Reflective-questions | **16**  Instructor-led  Film-instruction Web-education Reflective-questions Comp-feedback |

**The basic life support (BLS) training intervention:**1, Self-learning training with instructions from a mobile application (App-instruction); 2, Self-learning training with instructions from a mobile application and a preparatory web-based education; 3, Self-learning training with instructions from a mobile application and three reflective questions; 4, Self-learning training with instructions from a mobile application, a preparatory web-based education and three reflective questions; 5, Self-learning training with instructions from a video-film; 6, Self-learning training with instructions from a video-film and a preparatory web-based education; 7, Self-learning training with instructions from a video-film and three reflective questions; 8, Self-learning training with instructions from a video-film, a preparatory web-based education and three reflective questions; 9, Instructor-led training with instructions from a video-film (control-group); 10, Instructor-led training with instructions from a video-film and a preparatory web-based education
11, Instructor-led training with instructions from a video-film and three reflective questions; 12, Instructor-led training with instructions from a video-film, a preparatory web-based education and three reflective questions; 13, Instructor-led training with instructions from a video-film and feedback from a compression depth device;
14, Instructor-led training with instructions from a video-film, a preparatory web-based education and feedback from a compression depth device; 15, Instructor-led training with instructions from a video-film, three reflective questions and feedback from a compression depth device; 16, Instructor-led training with instructions from a video-film, a preparatory web-based education, three reflective questions and feedback from a compression depth device.

**The seven learning activities: The self-learning training** participants was encouraged to train according to the instructions, without an instructor, at work or at home with a personal training kit as many times as they wanted for about two weeks for practical training and for theoretical knowledge with no limit. The learning objective was included in the personal information for each participant; **The mobile-application instruction** was individually downloaded on a private or a workplace mobile phone or a mobile tablet. The mobile-application instruction lasted for about 30 minutes. It contained a short audio-visual information on sudden cardiac arrest (SCA) and the importance of to call 112, shout for an automated external defibrillator (AED), and start cardiopulmonary resuscitation (CPR) and use the AED, and instructions for how to perform CPR, use of the AED, briefly recovery position and foreign body airway obstruction (FBO); **The preparatory web-based education** contained theory and dramatized videos about symptoms of the most feared complications and treatment for cardiovascular disease (CVD) i.e. stroke, acute myocardial infarction (AMI, heart attack) and SCA and healthy lifestyle factors, and ten multiple-choice questions. The web-based education lasted for about 30 minutes. The platform was interactive and to proceed both correct answer on questions and click to continue was required. Feedback as text- and voice message and a certificate were generated by the platform; **The reflective questions** were three essential questions printed on a text document about the importance of to promptly call 112, hand placement for compressions and to perform BLS in a real-life out-of-hospital cardiac arrest (OHCA) situation. The instructor-led group discussed the questions in pair for about 15 minutes. The self-learning group was encouraged to reflect by their own or with a relative; **The film instruction** was from the standardised national digital video disc (DVD) for BLS training and lasted for 60 minutes. It contained a drama scenario from an OHCA situation, some theoretical knowledge on SCA and practical instructions for how to perform CPR and use of an AED, and briefly recovery position and FBO. The participants trained together with the video-instructor; **The instructor-led training** was organised in groups of 12-25 participants per one or two instructors by an independent coordinator at the workplace. The learning model was to practise on a personal training kit while watching an instruction film. The instructor introduced the learning activity for the group, helped the participants to achieve the learning objectives and answered the participants questions for about 15 minutes in addition to a film instruction. **The technical device for feedback on compression** depth was used in the instructor-led groups for about 15 minutes at the end of the course. The instructor facilitated the participants with the device placed on the manikin’s chest to achieve the correct compression depth.

**The equipment used in the intervention** was a personal Mini Anne manikin and a paperboard training AED in a kit (the Mini Anne kit, Laerdal Medical, Stavanger, Norway), the Mini Anne instruction film for BLS, CPR, AED, recovery position and FBO for 60 minutes (Swedish Resuscitation Council) and the mobile application instructions named “Save the Heart”, for 30 minutes (Swedish Resuscitation Council). For theoretical knowledge a preparatory interactive web-based education on cardio vascular disease (CVD), stroke, acute myocardial infarction (AMI), SCA, CPR, AED and healthy lifestyle factors called the “Help-Brain-Heart”, for about 30 minutes (Swedish Resuscitation Council), The equipment also comprised the “CPRmeter” (Laerdal Medical, Stavanger, Norway), which is a mechanical device for feedback on compressions, and three reflective essential questions on call for help, hand-position for compressions and willingness to act in a real-life OHCA situation, research information document, consent form and instructions for the individual training intervention (1-16).
