# Supplementary material for: Learning Outcome After Different Combinations of Seven Learning Activities in Basic Life Support on Laypersons in Workplaces: a Cluster Randomised, Controlled Trial
Source: Med Sci Educ. 2020 Nov 18;31(1):161–73. doi: 10.1007/s40670-020-01160-3 (PMC8368380; doi:10.1007/s40670-020-01160-3)
Supplement: Supplementary file 5 — (DOCX 21.6 kb) [file 40670_2020_1160_MOESM5_ESM.docx]

**Supplementary file 5, Table 6a. Theoretical knowledge on first action, stroke, AMI, healthy lifestyle factors, self-assessed theoretical knowledge and practical skills, confidence and willingness to act in a real-life OHCA situation – directly after training in BLS**

-------------------------------------------------------------------------------------------------------------------------------------------------

Control Intervention Intervention Intervention Intervention

9 10 11 14 16

(n=120) (n=122) (n=135) (n=155) (n=136)

------------------------------------------------------------------------------------------------------------------------------------------------

TOTAL SCORE FOR CORRECT ANSWERS

----------------------------------------------------

Theoretical knowledge of first action if

stroke or AMI, i.e. 112 (%) (1/0/0/0/0) * 98.3 97.5 100.0 100.0 98.5

Theoretical knowledge of first action if

OHCA, i.e. 112 (%) (1/0/0/2/1) 70.6 91.0 82.2 85.6 94.1

Theoretical knowledge of stroke

(mean±sd) (6/3/1/8/1) 4.3±2.0 5.6±1.6 4.2±2.1 5.6±1.7 5.9±1.3

Theoretical knowledge of AMI

(mean±sd) (7/1/5/5/4) 4.5±2.1 6.4±2.3 4.3±2.4 6.5±2.2 6.8±2.1

Theoretical knowledge of healthy lifestyle

factors (mean±sd) (10/1/5/5/1) 5.3±1.2 5.6±0.9 5.3±1.2 5.8±0.6 5.7±0.6

INDIVIDUAL SCORE FOR ANSWERED YES

-----------------------------------------------------

Self-assessed sufficient theoretical

knowledge and practical skills to be able to

perform compressions (%) (11/7/7/8/12) 99.1 98.3 99.2 96.6 100.0

Self-assessed sufficient theoretical

knowledge and practical skills to be able to

perform ventilations (%) (11/7/8/8/9) 100.0 98.3 99.2 98.0 100.0

Self-assessed sufficient theoretical

knowledge and practical skills to be able to

use an AED (%) (17/8/8/6/13) 98.1 96.5 99.2 97.3 100.0

Self-assessed confidence after training

(%) (8/3/3/4/4) 99.1 100.0 100.0 97.4 100.0

Self-assessed willingness to act if a relative

suffers an OHCA (%) (0/0/0/0/0)

Would not dare or want to intervene 0.0 0.0 0.7 0.6 0.0

Would give ventilations only 0.0 0.0 0.0 0.0 0.0

Would give chest impressions only 5.0 0.8 0.7 2.6 1.5

Would give both chest compressions

and ventilations 95.0 99.2 98.5 96.8 98.5

Self-assessed willingness to act if an unknown

person suffers an OHCA (%) (0/0/0/2/0)

Would not dare or want to intervene 2.5 1.6 0.0 2.0 2.2

Would give ventilations only 1.7 0.8 0.0 0.0 0.0

Would give chest impressions only 23.3 34.4 21.5 26.1 33.8

Would give both chest compressions

and ventilations 72.5 63.1 78.5 71.9 64.0

------------------------------------------------------------------------------------------------------------------------------------------------

* number of participants where information was missing in the five training groups, respectively

Control group number 9, instructor-led training and film-based instructions in BLS; 10, instructor-led training in BLS, film-based instructions and web-based education; 11, instructor-led training in BLS, film-based instructions and reflective questions; 14, instructor-led training in BLS, film-based instructions, web-based education and chest compression feedback device; 16, instructor-led training in BLS, film-based instructions, web-based education, reflective questions and chest compression feedback device.

Maximal point for each question was one point. The minimal and maximal score for first action was 0-1 point, for stroke 0-7 points, for AMI 0-9 points and for lifestyle factors 0-6 points

**Supplementary file 5, Table 6b. Theoretical knowledge on first action, stroke, AMI, healthy lifestyle factors, self-assessed theoretical knowledge and practical skills, confidence and willingness to act in a real-life OHCA situation – six months after training**

-------------------------------------------------------------------------------------------------------------------------------------------------

Intervention Intervention Intervention Intervention Intervention

9 10 11 14 16

(n=141) (n=115) (n=146) (n=157) (n=135)

------------------------------------------------------------------------------------------------------------------------------------------------

TOTAL SCORE FOR CORRECT ANSWERS

----------------------------------------------------

Theoretical knowledge of first action if

stroke or AMI, i.e. 112 (%) (2/0/0/0/0) * 99.3 100.0 100.0 98.7 100.0

Theoretical knowledge of first action if

OHCA, i.e. 112 (%) (3/0/0/2/1) 71.0 91.3 84.9 85.2 93.3

Theoretical knowledge of stroke

(mean±sd) (6/3/1/8/1) 4.3±2.0 5.6±1.6 4.2±2.1 5.6±1.7 5.9±1.3

Theoretical knowledge of AMI

(mean±sd) (7/1/5/5/4) 4.5±2.1 6.4±2.3 4.3±2.4 6.5±2.2 6.8±2.1

Theoretical knowledge of healthy lifestyle

factors (mean±sd) (10/1/5/5/1) 5.3±1.2 5.6±0.9 5.3±1.2 5.8±0.6 5.7±0.6

INDIVIDUAL SCORE FOR ANSWERED YES

-----------------------------------------------------

Self-assessed sufficient theoretical

knowledge and practical skills to be able to

perform compressions (%) (32/14/20/14/11) 98.2 99.0 98.4 97.2 100.0

Self-assessed sufficient theoretical

knowledge and practical skills to be able to

perform ventilations (%) (32/16/21/19/12) 98.2 100 98.4 97.1 98.4

Self-assessed sufficient theoretical

knowledge and practical skills to be able to

use an AED (%) (36/13/21/20/19) 96.2 94.1 98.4 96.4 99.1

Self-assessed confidence after training

(%) (16/11/8/7/2) 97.6 99.0 98.6 98.0 99.2

Self-assessed willingness to act if a relative

suffers an OHCA (%) (2/1/1/0/0)

Would not dare or want to intervene 0.7 0.0 1.4 0.0 0.0

Would give ventilations only 0.0 0.0 0.7 0.0 0.7

Would give chest impressions only 3.6 4.4 0.7 2.5 2.2

Would give both chest compressions

and ventilations 95.7 95.6 97.2 97.5 97.0

Self-assessed willingness to act if an unknown

person suffers an OHCA (%) (2/0/1/1/0)

Would not dare or want to intervene 4.3 1.7 0.7 2.6 2.2

Would give ventilations only 0.7 0.0 0.0 0.0 0.0

Would give chest impressions only 24.5 34.8 29.0 25.6 30.4

Would give both chest compressions

and ventilations 70.5 63.5 70.3 71.8 67.4

------------------------------------------------------------------------------------------------------------------------------------------------

* number of participants where information was missing in the five training groups, respectively

Control group number 9, instructor-led training and film-based instructions in BLS; 10, instructor-led training in BLS, film-based instructions and web-based education; 11, instructor-led training in BLS, film-based instructions and reflective questions; 14, instructor-led training in BLS, film-based instructions, web-based education and chest compression feedback device; 16, instructor-led training in BLS, film-based instructions, web-based education, reflective questions and chest compression feedback device.

Maximal point for each question was one point. The minimal and maximal score for first action was 0-1 point, for stroke 0-7 points, for AMI 0-9 points and for lifestyle factors 0-6 points
